# Cross-cultural adaptation of an implementation science glossary into simplified Chinese: study protocol

**DOI:** 10.3389/frhs.2026.1799760

**Published:** 2026-04-07

**Authors:** Xiaomeng Ye, Wyatt Xixuan Wu, Tina Yen-Ting Chen, Qian Yang, Nick Sevdalis

**Affiliations:** 1 Centre for Behavioural and Implementation Science Interventions, Yong Loo Lin School of Medicine, National University of Singapore, Singapore; 2 School of Public Health and the Department of Geriatrics, the Fourth Affiliated Hospital, and International School of Medicine, Zhejiang University School of Medicine, Zhejiang University, Hangzhou, China

**Keywords:** Chinese language, cross-cultural adaptation, glossary, methodological study, terminology, translation

## Abstract

**Introduction:**

Implementation science (IS) relies on standardized terminology, yet existing glossaries are largely English and Western-centric, creating risks of misinterpretation in other contexts. In China, with over one billion Chinese speakers and a rapidly expanding IS community, the absence of a unified glossary hinders training and knowledge exchange. We report a prospective, multi-stage cultural and linguistic adaptation study of the Implementation Science Research Glossary produced by the Centre for Implementation Science at King's College London, UK, from its original English version into Simplified Chinese.

**Methods:**

This study follows an established cross-cultural adaptation framework, modified to reflect the nature of a glossary rather than a psychometric instrument. The process includes: (1) forward translation of the English glossary into Simplified Chinese by a bilingual translator with IS expertise; (2) independent back translation by another bilingual individual blinded to the original glossary; (3) structured reconciliation involving the forward translator, back translator, and an additional reviewer with IS knowledge; (4) first expert panel review—conducted by 5 experts in IS and public health, who will also participate in the final review—to assess semantic, idiomatic, experiential, and conceptual equivalence, leading to a refined version of the glossary; consensus will be defined *a priori* as ≥80% agreement on each term and definition (5) evaluation of the refined glossary through two complementary quantitative validation procedures: (a) content validation by 6–10 eligible faculty members using the Content Validity Index (CVI), and (b) response process validation by 10–30 Chinese-speaking postgraduate students using the Face Validity Index (FVI); items will be considered acceptable if I-CVI ≥0.83 and glossary-level S-CVI/Ave ≥0.90 for content validity, and I-FVI ≥0.80 with glossary-level S-FVI/Ave ≥0.90 for face validity; and (6) final expert panel review to reach consensus on the adapted glossary. Ethical approval will be obtained prior to data collection.

**Discussion:**

The study will produce the first, to our knowledge, culturally and linguistically adapted IS glossary for Chinese-speaking contexts. This resource is expected to enhance clarity and accessibility of implementation concepts, supporting research and practice in local settings. The documented adaptation process will provide a methodological reference for future translation of IS resources in other languages.

## 1 Introduction

Implementation science (IS) is a field of research concerned with understanding and improving the processes by which evidence-based practices are adopted, implemented, and sustained in real-world settings, particularly within health care systems (1). Rather than focusing on the development of new clinical evidence, IS seeks to address the persistent gap between what is known to be effective and what is routinely delivered in practice. Central objectives of the field include identifying determinants of implementation success and failure, designing and evaluating implementation strategies, and generating generalizable knowledge about how to promote high-quality, evidence-informed care across diverse contexts (1, 2).

Over the past three decades, IS has been applied extensively in health care, where it has informed efforts to improve adherence to clinical guidelines, reduce unwarranted variations in care, enhance quality improvement initiatives, and strengthen the delivery of complex interventions at individual, organizational, and system levels (1, 3). The field draws on theories from behavioral science, organizational studies, and health services research to examine the complex and multilevel determinants of implementation processes (1, 2). As a result, IS encompasses a wide range of constructs, frameworks, and methodological approaches, contributing to the rapid expansion and diversification of its terminology.

The increasing diversity of terminology in IS has heightened the importance of shared and clearly defined concepts. Inconsistent or overlapping use of key terms may reduce interpretability, complicate comparison across studies, and limit the accumulation of coherent knowledge within the field (4). Standardized terminology therefore plays a foundational role in facilitating collaboration between researchers, practitioners, and policymakers, enabling shared understanding of concepts such as implementation strategies, determinants, and outcomes (5). To address this need, there has been a recent trend in developing IS glossaries. These include, for example, the Implementation Science Research Glossary produced by the Centre for Implementation Science at King's College London in the UK (6), the Glossary of Key Implementation Science Terms and Acronyms developed by Implementation Scientists, LLC and Wilson Language Training (7), and the Dissemination & Implementation Glossary maintained by the University of Colorado Anschutz Medical Campus (8). These glossaries aim to harmonize language across research and practice, improving training and guiding effective implementation efforts.

One shared aspect of the glossaries currently available is that they are developed in English and informed by Western healthcare systems and cultural paradigms. This is not surprising, given the history and developmental trajectory of IS, which has largely taken place in Anglo-Saxon countries. While valuable, direct application of such glossaries in other linguistic and cultural contexts can be problematic (4). Direct translation of IS terminology often results in semantic ambiguity, loss of conceptual precision, and cultural incongruence (9). Beyond linguistic inaccuracy, translation without systematic cross-cultural validation may alter construct boundaries and implicit theoretical assumptions embedded within key IS concepts, thereby threatening construct validity and theoretical coherence (10, 11). Key IS terms—such as “fidelity,”“engagement,” and “involvement”—lack direct equivalents in many languages, including Chinese; or carry different cultural, philosophical and practical connotations. Such discrepancies risk conceptual misinterpretation, weaken training, and constrain the uptake of IS frameworks in emerging research contexts (12). Over time, inconsistent interpretation and operationalization of core terminology may lead to variability in measurement, reduced comparability across studies, and superficial or distorted application of IS frameworks (13–15). These challenges are compounded by differences in healthcare systems and academic traditions. In China, where IS is rapidly expanding yet terminological standards remain underdeveloped (16), the absence of a culturally adapted glossary presents a challenge in knowledge transfer and local advancement. Without culturally adapted language, knowledge transfer may be inconsistent, hindering both research rigor and practical application (12). Given the scale and growing influence of the Chinese-speaking research community, failure to establish standardized and conceptually equivalent terminology may limit meaningful international collaboration and cumulative knowledge development. Accordingly, developing a unified Simplified Chinese glossary has the potential to enhance methodological coherence, accelerate capacity building, and generate substantial international impact.

Systematic, evidence-based approaches to support cross-cultural standardization of the terms used within a particular field of research are available. Cross-cultural adaptation frameworks, such as the guidelines developed by Beaton et al. (10) and Brislin's back-translation model (17), have been developed over 20 years ago and have been widely applied to the adaptation of patient-reported outcome measures and other health-related measurement instruments. These frameworks emphasize achieving semantic, idiomatic, experiential, and conceptual equivalence through systematic, well-defined steps, including forward translation, back translation, expert committee review, and pretesting.

Forward translation aims to produce an initial version that reflects the intended meaning of the source text while accounting for linguistic conventions in the target language. Back translation serves as a verification step to identify discrepancies or unintended shifts in meaning by comparing the translated version with the original source. Expert committee review allows multidisciplinary evaluation of problematic terms and ensures that culturally embedded assumptions and contextual nuances are appropriately addressed. Pretesting, including cognitive review or pilot assessment, helps detect ambiguities, misinterpretations, or unintended connotations among potential end users. Together, these procedures move beyond literal translation and provide a structured mechanism for safeguarding both linguistic accuracy and conceptual integrity (10, 17).

While originally designed for psychometric tool adaptation, their principles are applicable to conceptual resources, such as the IS glossaries that we focus on here. To the best of our knowledge, application of any such established framework to translate IS terminology has not been undertaken. Existing efforts to translate or adapt glossaries often rely on *ad hoc* processes without methodological transparency, which increases the risk of inconsistent terminology and conceptual misalignment (18). Here we argue that adopting a structured and transparent adaptation process is essential to support methodological rigor and reproducibility in the development of IS terminology across contexts.

The objective of this study is to develop, for the first time, a culturally and linguistically adapted IS glossary in Simplified Chinese using a structured cross-cultural validation process to ensure semantic and conceptual equivalence for Chinese-speaking academic and healthcare settings. The resulting glossary is intended not only to provide a standardized terminology resource for IS stakeholders, but also to demonstrate a transparent and replicable methodological approach to terminology adaptation in emerging implementation contexts.

## 2 Methods

### 2.1 Study design

This study employs a cross-cultural adaptation design, drawing on the frameworks proposed by Beaton et al. (10) and Brislin's back-translation model as adapted by Jones et al. (17). Recognizing that glossaries are conceptual reference tools rather than psychometric instruments, the process has been adapted accordingly. The adaptation will involve sequential stages of translation, reconciliation, expert review, and quantitative validation through content validation (via a Content Validity Index survey) and response process validation (via a Face Validity Index survey) (19–23), culminating in a final expert panel consensus to produce the Chinese version of the glossary.

### 2.2 IS glossary to adapt

The glossary selected for adaptation is the Implementation Science Research Glossary developed by the Centre for Implementation Science at King's College London, as part of the National Institute for Health and Care Research (NIHR) Applied Research Collaboration (ARC) South London (2024), UK (6). At the time the study was conceived, this glossary represented one of the most recent and comprehensive resources dedicated specifically to IS terminology, to our knowledge. It was designed as an open-access resource to provide clear, accessible, and standardized definitions of key terms in IS for researchers, practitioners, and policymakers. The glossary contains over 100 terms and acronyms commonly used in the field, each accompanied by definitions and references where relevant, thereby offering both breadth of coverage and transparency regarding the conceptual sources underpinning each definition. As a reference resource, it aims for greater consistency in terminology, supports communication across disciplines, and offers a foundation for training and capacity development in implementation research and practice. Compared with other available glossaries, which may focus on selected subdomains (e.g., dissemination or knowledge translation) or provide more limited term coverage (7, 8), this glossary was considered particularly suitable for cross-cultural adaptation due to its field-wide scope, contemporary relevance, and explicit effort to harmonize terminology across research and applied health settings.

### 2.3 Participants

The study will involve three categories of participants: (1) a translation and reconciliation team responsible for the initial adaptation of the glossary, (2) an expert review panel tasked with evaluating the quality and conceptual equivalence of the adapted version, and (3) faculty members and postgraduate students in IS who will contribute to the validity assessment of the glossary.

The translation and reconciliation team will consist of three individuals with complementary expertise. The forward translator will be a native Chinese speaker (XY) with formal MSc-level training in IS and demonstrated fluency in English, tasked with producing a conceptually faithful Simplified Chinese version of the glossary. The back translator (XW) is also a native Chinese speaker, fluent in English and with formal MSc-level training in IS, who will independently translate the Chinese version back into English while remaining blinded to the original text to reduce potential bias. To enhance the validity and rigor of the reconciliation process, a third member (NS), a senior implementation scientist, will participate in the structured comparison of the original and back-translated versions. During reconciliation, all three members will review the translated glossary aiming to identify and resolve discrepancies. The reconciliation will be carried out initially by XY and XW, with NS reviewing as a third reviewer and providing conceptual and theoretical clarification from an IS standpoint. All decisions will be reached through reasoned consensus, and detailed records of discussions, rationales, and final choices will be maintained in a version-controlled reconciliation log to ensure transparency and reproducibility.

The expert review panel will comprise five professionals with demonstrable expertise in IS, public health, health policy, or related applied health research fields. All authors will serve on the panel; if required, additional experts will be identified through purposive and snowball sampling strategies, leveraging academic networks and professional affiliations to ensure disciplinary diversity and cultural insight. Most members will be bilingual (XY, XW, TC, QY), with proficiency in both English and Simplified Chinese, enabling them to evaluate technical and conceptual equivalence across linguistic and cultural contexts. The panel will include NS, who will provide expert input on conceptual clarity, theoretical accuracy, and consistency with IS terminology. The expert panel will be engaged at two different phases of the study: firstly to review the reconciled translation prior to validity assessment, and secondly to finalize consensus following the validity assessment process.

To further strengthen the rigor of the adaptation, the validation phase will involve two complementary groups of participants recruited through purposive and snowball sampling. For content validation, 6–10 Chinese-speaking faculty members in IS will be invited to complete a Content Validity Index (CVI) survey of the refined glossary (see next section for details on the CVI process). Recruitment eligibility will be defined as either (1) a minimum of five years of experience in a faculty position with demonstrable engagement in IS or implementation research, or (2) at least five peer-reviewed publications in IS or implementation research, thereby ensuring established academic engagement in the field. These faculty participants will evaluate the relevance and domain appropriateness of each glossary term and definition.

For response process (face) validation, at least 10 and ideally up to 30 Chinese-speaking postgraduate students in IS will be recruited to complete a Face Validity Index (FVI) survey (see next section for details on the FVI process). Postgraduate participants will assess the clarity, comprehensibility, and interpretability of the translated terminology from a target-user perspective.

All participants will be invited to contribute based on their language and subject matter expertise and interest in the topic, and they will provide informed consent before participating. Participants will engage with the study either in person or via secure online platforms, depending on their location and availability.

### 2.4 Procedures

The adaptation will be conducted in six stages, as per the frameworks used to guide the study (10, 17):

**Stage 1:** In the *forward translation stage*, the glossary will be translated from English to Simplified Chinese by a bilingual translator (XY) with IS expertise. Emphasis will be placed on preserving conceptual meaning rather than producing literal translations.

**Stage 2:** The *back translation* will be performed by an independent bilingual translator (XW) blinded to the original English glossary. This process is designed to detect discrepancies or distortions in meaning introduced during forward translation.

**Stage 3:** During the *reconciliation stage*, the forward translator (XY), back translator (XW), and third reviewer (NS) will conduct a structured comparison of the original glossary, the translated version, and the back-translated version. Discrepancies will be discussed and resolved through consensus, with all three participants’ perspectives given equal weight. In instances where literal translations are conceptually accurate but potentially ambiguous or unnatural for Chinese readers, authoritative Chinese-language IS textbooks and reference materials will be consulted [e.g., Implementation Science in Health (24)]. When standardized translations of relevant terms are identified in these sources, they will be adopted where appropriate to enhance terminological consistency and conceptual clarity across the field. All decisions, rationales, and final choices will be documented in a version-controlled reconciliation log to ensure methodological transparency and reproducibility.

**Stage 4:** Following reconciliation, an *expert panel* of 5 professionals with relevant expertise (all study authors) will convene in a synchronous joint discussion session conducted via a secure online platform to evaluate the glossary for semantic, idiomatic, experiential, and conceptual equivalence. The session will follow a structured modified nominal group technique process (25) designed to ensure balanced participation and systematic consensus building.

Each term and its corresponding definition will be reviewed sequentially. Panel members will first provide independent reflections to minimize early conformity effects, followed by structured round-robin discussion to clarify interpretations and propose revisions. Proposed translations will then be evaluated through explicit confirmation of agreement. Consensus will be defined *a priori* as at least 80% of panel members (i.e., four or more members) indicating satisfaction with the proposed wording.

When disagreement persists after discussion, alternative translations will be documented and subsequently evaluated during the quantitative validation stage (see Stage 5). If necessary, additional independent experts may be consulted to avoid decision-making deadlock. Feedback from this first panel review will be used to refine the glossary before proceeding to the quantitative assessment of the next stage.

**Stage 5:** The refined glossary will undergo two complementary quantitative validation procedures designed to generate content and response process validity evidence.

**Stage 5a: Content Validation**

Six to ten eligible faculty members in IS will complete a *CVI* survey (see Section 2.3 for eligibility criteria). Faculty participants will independently rate the relevance and domain appropriateness of each translated term and definition. Item-level CVI (I-CVI) and scale-level CVI (S-CVI) statistics will be calculated to identify terms requiring revision or further discussion (19). In the present study, the S-CVI will be interpreted as a glossary-level index reflecting the overall content validity of the adapted terminology set.

**Stage 5b: Response Process (Face) Validation**

At least 10 and ideally up to 30 Chinese-speaking postgraduate students in IS will complete a *FVI* survey. Participants will evaluate the clarity, comprehensibility, and interpretability of each translated term and definition. Item-level FVI (I-FVI) and scale-level FVI (S-FVI) indices will be calculated to identify entries that, while conceptually appropriate, may be linguistically unclear or difficult to interpret from a user perspective (22). In the present study, the S-FVI will be interpreted as a glossary-level index reflecting the overall clarity and interpretability of the adapted terminology set.

**Stage 6:** Following completion of the CVI and FVI analyses, the expert panel will reconvene in a structured joint session to review quantitative findings and address any remaining issues (including items carried forward from Stage 4 where consensus cannot be reached). Terms identified as problematic in either validation stage will be discussed and revised through iterative consensus. The final adapted glossary will be established once agreement is achieved across all panel members.

**Figure 1:**
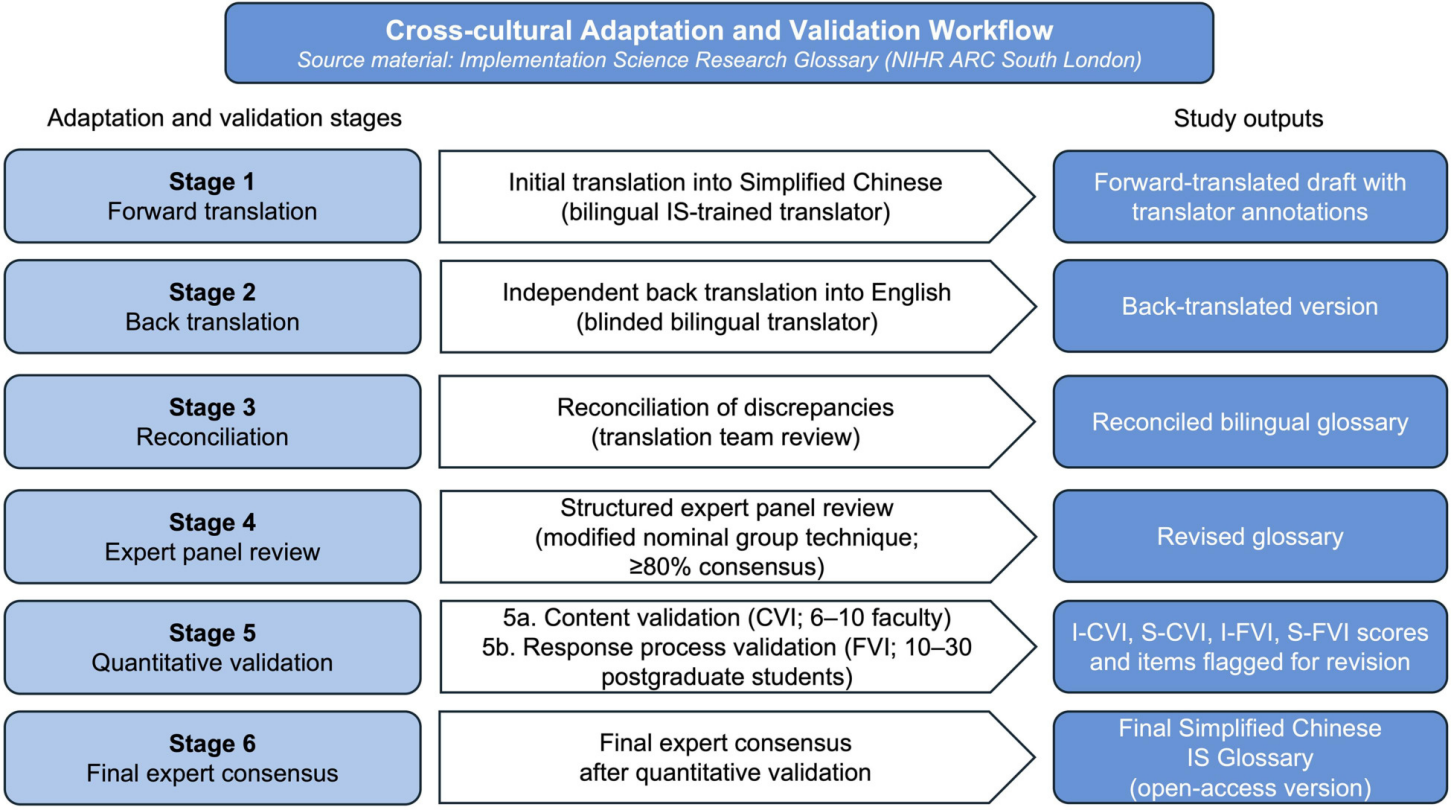
Cross-cultural adaptation and validation workflow for the implementation science glossary. IS, implementation science; CVI, content validity index; FVI, face validity index; I-CVI, item-level content validity index; I-FVI, item-level face validity index; S-CVI, scale-level content validity index; S-FVI, scale-level face validity index.

Throughout the study, decision-making will be systematically documented to ensure transparency and to provide a methodological reference for future adaptation of IS resources in other linguistic and cultural settings. The full workflow of the planned adaptation process is illustrated in Figure 1.

### 2.5 Data management and analysis

Data management will focus on the systematic documentation and secure handling of all materials generated throughout the adaptation process. All glossary versions—including the original, forward translation, back translation, reconciled drafts, and subsequent revisions—will be securely stored and version-controlled to preserve a clear audit trail.

During reconciliation and expert panel review, detailed records of adaptation choices and their justifications will be maintained to support internal consistency and transparency. All proposed revisions, comments, and alternative translations will be documented directly within a shared cloud-based document, where panel members can annotate, suggest edits, and provide written comments. In addition, expert panel discussions conducted via secure online sessions will be audio-recorded, and key points, areas of disagreement, and agreed revisions will be summarized after each session. Decisions will be reached through the predefined consensus criteria described in Stage 4, and all finalized adaptations will be logged in a version-controlled reconciliation document to ensure traceability and methodological transparency.

For the CVI analysis, items will be considered acceptable if the I-CVI is ≥0.83 when rated by six or more faculty members, consistent with established recommendations for content validation. The S-CVI based on the average method (S-CVI/Ave), interpreted here as a glossary-level index, will be considered satisfactory if ≥0.90 (19).

For the FVI analysis, items will be considered acceptable if the I-FVI is ≥0.80, in line with recommended thresholds for response process validation involving ten or more raters. The glossary-level S-FVI/Ave will be considered satisfactory if ≥0.90 (22).

Items failing to meet the predefined I-CVI or I-FVI thresholds will be flagged for revision. The expert panel will review all flagged entries in Stage 6, taking into account quantitative indices and qualitative feedback. Where revisions are made, the panel will determine through structured discussion whether the adapted wording adequately addresses identified concerns. Final decisions will be reached through consensus, and removal of a term will only be considered if the panel concludes that meaningful adaptation is not feasible without compromising conceptual integrity.

All digital materials will be stored on encrypted, access-restricted platforms. Any identifying participant information will be anonymized unless required as part of the study process and explicit written permission is provided. Access to raw data and revision logs will be restricted to the core research team.

### 2.6 Ethical considerations

Ethical approval for this study has been obtained from the Institutional Review Board (IRB) at the National University of Singapore (NUS) prior to initiating any data collection activities (IRB Ref No.: NUS-IRB-2025-878; Approved on 25 November 2025). All participants involved in the translation, reconciliation, expert review stages and the quantitative survey will be provided with detailed information about the study's purpose, procedures, voluntary nature of participation, and confidentiality protections. Written informed consent will be obtained from each participant before their involvement begins. All interactions with participants, whether conducted in person or remotely, will adhere to principles of ethical research conduct, including respect for autonomy, transparency, and the right to withdraw at any point without penalty.

Data will be stored on secure, access-restricted digital platforms managed by the research team. Any potentially identifying information, such as names or affiliations of expert panel members, will be anonymized in research records and final publications unless explicit written permission is granted. The study will comply with the NUS guidelines on data protection and research integrity.

## 3 Discussion

The globalization of IS has generated growing interest in applying its conceptual tools across linguistic and cultural boundaries. However, the translation of specialized terminology remains a neglected methodological challenge. Core concepts in IS, such as “fidelity,” “adaptation,” or “context”, are shaped by theoretical assumptions and cultural norms that do not always have direct equivalents in other languages or systems (16, 26). Without careful adaptation, such translations risk distorting meaning, introducing conceptual drift, or failing to resonate with local stakeholders.

Translation in cross-cultural research is not merely a linguistic exercise but a process of establishing semantic, conceptual, and content equivalence across sociocultural contexts (27, 28). Literal or unstructured translation risks construct nonequivalence, whereby lexical similarity masks shifts in theoretical meaning or practical implication. Such shifts threaten internal validity and undermine cross-context comparability (28).

Accordingly, methodological scholarship emphasizes that forward translation alone is insufficient. Instead, rigorous cross-cultural adaptation requires independent translation, back-translation, multidisciplinary review, and structured validation to ensure semantic and conceptual equivalence (27, 28). These procedures help preserve the theoretical boundaries of constructs when they are introduced into a different linguistic and disciplinary environment. Without such safeguards, conceptual distortions may remain undetected, resulting in findings that reflect linguistic misalignment rather than substantive contextual differences (28).

The importance of such methodological rigor is particularly salient in contexts where IS is still consolidating its disciplinary foundations. Recent empirical work suggests that IS in Asian settings faces persistent barriers, including limited conceptual familiarity, insufficient training opportunities, and low awareness of IS terminology and frameworks (29). In parallel, emerging implementation trials in China increasingly draw on established IS frameworks and theories (14), reflecting growing engagement with the field's theoretical architecture. However, the uptake of these concepts within Chinese-speaking systems depends not only on access to translated materials, but on the availability of terminological resources that accurately convey their conceptual scope and epistemological assumptions. In this sense, culturally robust translation is not merely a technical exercise but a foundational step in enabling coherent knowledge transfer and disciplinary development across linguistic contexts.

The present study operationalizes these methodological principles through a structured and transparent adaptation process, seeking to balance conceptual fidelity with contextual intelligibility. The study positions glossary development as part of capacity development work necessary for growing and sustaining IS scholarship across linguistic boundaries. The research aims to contribute to the field of IS in two main respects. Firstly, it advances a cross-cultural adaptation methodology which we have developed for a conceptual reference tool, rather than a psychometric instrument; the latter have traditionally been the focus of this literature (10). This distinction is important because glossaries, unlike surveys or measures, are not intended to capture latent traits or produce standardized scores, but to facilitate shared understanding. Prior efforts to localize health policy or social science terminology have often relied on informal or undocumented translation processes, leading to inconsistent use across texts and institutions (30). By applying a multi-stage adaptation framework—including blinded back translation, multi-expert reconciliation, structured review, and quantitative assessment—this study provides a replicable template that addresses both linguistic and epistemological equivalence for such tools.

Secondly, the study advances the idea that terminology translation should be understood as both a linguistic and epistemological task. In IS, many constructs are embedded within institutional, organizational, and policy contexts that differ across settings (5, 26). Translation, therefore, involves more than identifying lexical equivalents; in addition, it requires ensuring that concepts remain meaningful, relevant, and actionable within local systems. This study demonstrates how structured and transparent procedures, combining expert input with systematic evaluation, can help safeguard conceptual fidelity while accommodating contextual variation. By engaging multiple forms of expertise and documenting decision-making processes, the study positions translation as a mechanism for knowledge transfer that upholds the fidelity of source concepts while facilitating their effective application in the target context. This perspective aligns with broader calls in global health and social sciences to move beyond simple linguistic equivalence and toward methodologies that explicitly recognize the interplay of language, culture, and knowledge systems (28).

This study will be subject to limitations. The glossary is based on existing English-language definitions and reflects the epistemological assumptions embedded within them. While the adaptation process improves accessibility, it does not critically interrogate whether all constructs are equally relevant or transferable across systems, which is beyond the scope of the present methodology and would require broader empirical and theoretical work in local contexts. The inclusion of CVI and FVI assessments provides a structured and quantifiable means of assessing content (faculty-based) and response process/face validity (postgraduate user-based) evidence, but the evaluation is limited to the judgments of a relatively small panel of raters. Although the participants will consist of professionals with relevant disciplinary expertise, their perspectives will likely not encompass the full diversity of views across all Chinese-speaking regions or subfields within IS. In addition, selection bias and expert homogeneity remain possible, as faculty and expert contributors are likely to be academically trained and already familiar with established IS frameworks, which may privilege particular theoretical perspectives and disciplinary norms. As a result, the adapted glossary may reflect a consensus among IS-informed academic stakeholders more strongly than the interpretive perspectives of frontline practitioners, policymakers, or users working in varied institutional settings across Chinese-speaking contexts. Besides, the study does not incorporate a formal field pretest, meaning that downstream user validation, such as integration into graduate teaching, research collaborations, or stakeholder workshops, remains a necessary next step to confirm the glossary's practical utility and broader applicability by IS research and practice groups in Chinese-speaking settings.

Despite these limitations, this study offers both a practical product in the form of a vetted Simplified Chinese IS glossary and a methodological contribution that may guide future adaptation efforts in other languages and disciplinary contexts. Although the present work focuses on Simplified Chinese and IS, the adaptation process applied has wider application. Several academic fields rely on specialized conceptual vocabularies developed in English language contexts, while large linguistic communities remain underrepresented in research production or science standardization. The methodological principles of this study may, therefore, be applicable to other languages and to other disciplines characterized by dense theoretical terminology. In contexts where conceptual clarity and shared language are essential to research collaboration and policy translation, such glossaries can serve as foundational infrastructure. In practical terms, the glossary could be integrated into graduate and continuing professional education to support standardized instruction of IS concepts, incorporated into research collaboration workflows (e.g., shared terminology in research protocols and reporting templates), and referenced in implementation planning and policy-facing documents to improve conceptual clarity among multidisciplinary stakeholders. Future work might explore co-creation models that involve users earlier in the adaptation process, or examine how glossary use influences knowledge uptake, training, and interdisciplinary communication. To evaluate real-world impact, future studies could assess changes in learners’ conceptual understanding following glossary-supported training, examine the consistency of terminology use in publications or implementation reports produced by Chinese-speaking teams, and explore stakeholder perceptions of usability and relevance in applied settings. Such ‘downstream’ evaluation would help determine whether terminological standardization translates into more coherent communication, improved reporting quality, and stronger cumulative knowledge development over time.
